# Air Pollution Particulate Matter Amplifies White Matter Vascular Pathology and Demyelination Caused by Hypoperfusion

**DOI:** 10.3389/fimmu.2021.785519

**Published:** 2021-11-16

**Authors:** Mikko T. Huuskonen, Qinghai Liu, Krista Lamorie-Foote, Kristina Shkirkova, Michelle Connor, Arati Patel, Axel Montagne, Hans Baertsch, Constantinos Sioutas, Todd E. Morgan, Caleb E. Finch, Berislav V. Zlokovic, William J. Mack

**Affiliations:** ^1^ Zilkha Neurogenetic Institute, University of Southern California, Los Angeles, CA, United States; ^2^ Department of Neurosurgery, Washington University School of Medicine, St. Louis, MO, United States; ^3^ Department of Neurological Surgery, University of California San Francisco School of Medicine, San Francisco, CA, United States; ^4^ Department of Civil and Environmental Engineering, Viterbi School of Engineering, University of Southern California, Los Angeles, CA, United States; ^5^ Leonard Davis School of Gerontology, University of Southern California, Los Angeles, CA, United States; ^6^ Department of Neurological Surgery, Keck School of Medicine, University of Southern California, Los Angeles, CA, United States

**Keywords:** air pollution, blood brain barrier, hypoperfusion, MRI, carotid artery stenosis

## Abstract

Cerebrovascular pathologies are commonly associated with dementia. Because air pollution increases arterial disease in humans and rodent models, we hypothesized that air pollution would also contribute to brain vascular dysfunction. We examined the effects of exposing mice to nanoparticulate matter (nPM; aerodynamic diameter ≤200 nm) from urban traffic and interactions with cerebral hypoperfusion. C57BL/6 mice were exposed to filtered air or nPM with and without bilateral carotid artery stenosis (BCAS) and analyzed by multiparametric MRI and histochemistry. Exposure to nPM alone did not alter regional cerebral blood flow (CBF) or blood brain barrier (BBB) integrity. However, nPM worsened the white matter hypoperfusion (decreased CBF on DSC-MRI) and exacerbated the BBB permeability (extravascular IgG deposits) resulting from BCAS. White matter MRI diffusion metrics were abnormal in mice subjected to cerebral hypoperfusion and worsened by combined nPM+BCAS. Axonal density was reduced equally in the BCAS cohorts regardless of nPM status, whereas nPM exposure caused demyelination in the white matter with or without cerebral hypoperfusion. In summary, air pollution nPM exacerbates cerebrovascular pathology and demyelination in the setting of cerebral hypoperfusion, suggesting that air pollution exposure can augment underlying cerebrovascular contributions to cognitive loss and dementia in susceptible elderly populations.

## Introduction

Cerebrovascular dysfunction is associated with dementia with anatomical damage to white matter (1). Notably, brain hypoperfusion is associated with multiple forms of dementia and white matter structural changes (2, 3). Environmental and lifestyle factors modify the risk of neurodegenerative diseases. Population-based studies strongly associate elevated levels of air pollution particulate matter with reduced brain white matter regional volumes (4–8). Additionally, experimental studies with mice show harmful effects of particulate matter (PM) on both corpus callosum white matter and hippocampus (7–10). The joint effects of underlying health conditions and environmental exposures are understudied. In the current experiments, we investigate the effect of nanoparticulate matter (nPM, aerodynamic diameter ≤200 nm) on brain white matter pathology in a murine bilateral carotid artery stenosis (BCAS) model of chronic cerebral hypoperfusion (CCH) which has been previously shown to induce white matter injury (11). The outcome measures include translational *in vivo* magnetic resonance imaging (MRI) and histological markers to characterize vascular and structural white matter pathologies (12). We hypothesize that nPM exposure amplifies the effects of CCH on white matter injury and blood brain barrier (BBB) permeability.

## Materials and Methods

### Animals

**Figure 1:**
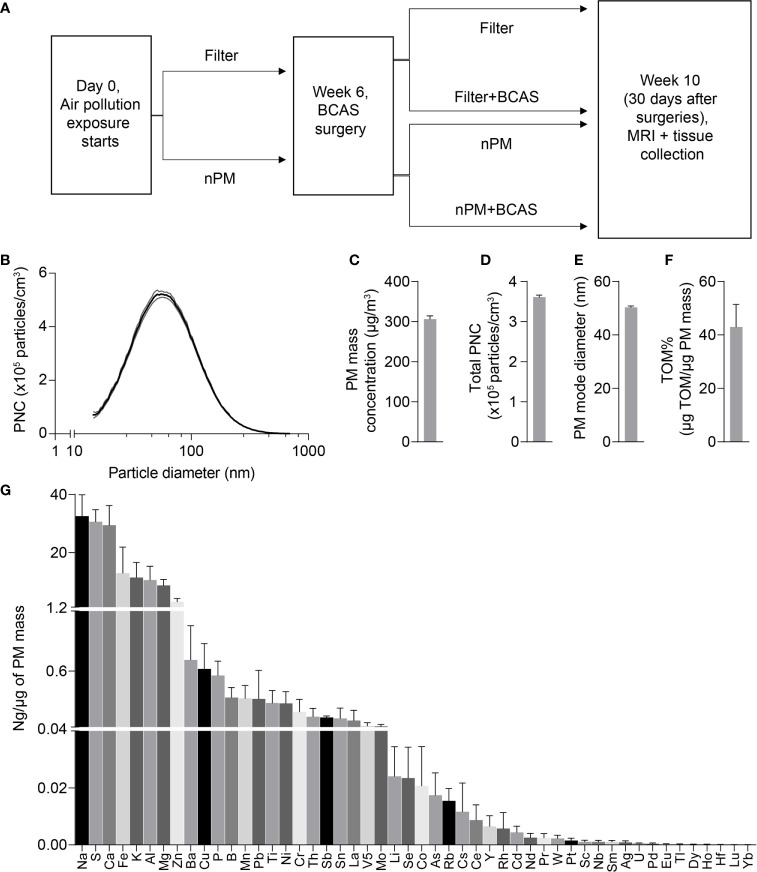
Design of the study and physicochemical properties of nPM used in exposure studies. **(A)** Experimental timeline of air pollution exposure and surgical procedures. **(B)** Particle size distribution of the collected exposure aerosol. **(C)** Particulate matter mass concentration (µg/m^3^), **(D)** Total particulate number concentration (x10^5^ #/m^3^), **(E)** Particle mode diameter (nm), **(F)** Total mass of organic matter (% of particulate matter mass) and **(G)** Mass fractions of trace elements and metals (ng/µg of particulate matter mass) during exposures. Data is presented as mean ± standard deviation in panel **(B)** and mean ± standard error in panels **(C–H)**. (n)PM, (nano)particulate matter; BCAS, bilateral carotid artery stenosis; MRI, magnetic resonance imaging; PNC, particulate number concentration; TOM, total mass of organic material.

All procedures were reviewed and approved by the Institutional Animal Care and Use Committee at the University of Southern California. Male C57BL/6J mice were purchased from Jackson Laboratories and used at 8-10 weeks of age (24-29 g, at the beginning of the exposures). Animals were housed under USC Department of Animal Resources on a 12-hour light dark cycle with free access to food and water (except during the nPM/filtered air exposure periods). Mice were group housed with four mice per cage and single mice were randomized to one of four exposure groups: 1) filter 2) nPM 3) filter+BCAS 4) nPM+BCAS (**Figure 1A**).

### Nanoparticulate Matter (nPM) Exposure

nPM (particulate matter smaller than 0.2 μm in diameter) was collected in an urban area in central Los Angeles in direct proximity to a busy roadway, primarily impacted by traffic emissions (13, 14). Briefly, nPM was collected at 400L/min flow using a high-volume ultrafine particle sampler that removes particles larger than 0.2 μm by means of a multiple rectangular (slit) geometry jet conventional impactor. The resulting nPM was collected on pretreated Teflon filters (8x10”, PTFE, 2 μm pore) placed directly downstream of the impactor and transferred into an aqueous suspension by soaking in Milli-Q deionized water (total organic compounds <10 ppb, particle free, endotoxin <1units/mL, endotoxin-free glass vials) for 30min. Per US EPA recommended procedures, aqueous nPM suspensions were stored at -20°C (15).

The nPM suspensions were re-aerosolized by means of a HOPE jet nebulizer (Model 11310, B&B Medical Technologies, Carlsbad, CA, USA) as described in greater detail in (13). A total of 15L/min of aerosol flow was generated, with the majority (10L/min) drawn through the exposure chambers, and the remaining 5L/min diverted to Teflon and quartz filters for particle collection and characterization. The mass concentration of nPM was determined by pre- and post-weighing the Teflon filter under controlled temperature and relative humidity. Inorganic ions [ammonium (NH_4_^+^), nitrate (NO_3_^-^), and sulfate (SO_4_^2-)^] were analyzed by ion chromatography. PM-bound metals and trace elements were analyzed by magnetic-sector inductively coupled plasma mass spectroscopy. Water-soluble organic carbon on the quartz filter was analyzed by GE-428 Sievers liquid analyzer (GE-Sievers, Boulder, CO).

Mice were exposed to either re-aerosolized nPM or filtered air for five hours per day, three days per week for 10 weeks (150 total hours) (**Figure 1A**). Exposures were conducted in temperature and air controlled sealed whole-body chambers with adequate ventilation. Different cohorts of mice were used for MRI (2 cohorts) and Immunohistochemistry (2 cohorts). Particle characteristics from all cohorts were averaged and are presented in **Figure 1**. **Table 1** demonstrates the particle characteristics for every nPM batch used in this study. **Table 2** lists the mass fractions of organic carbon, trace elements, and metals used in each nPM batch. Histochemistry studies were performed on stored tissue samples from mice exposed to nPM in 2015-2016 using nanoparticles collected in 2015 and 2016. MRI studies were performed on mice exposed to nPM in 2018 using nanoparticles collected in 2018 (batch 6,7, **Table 1**) (18).

**Table 1:** Exposure number and data analysis.

Exposure Number	Results	nPM Collection Dates	nPM Exposure Dates	PM Mass concentration (ug/m^3^)*^a^*	Total PNC (#/cm^3^)*^b^*	PN mode diameter (nm)	TOM% (µg TOM/µg PM mass)*^c^*	Mortality Rate (%)
1*^d^ (batch 2015a)^e^*	IHC	Oct-Dec 2015	2015	330 ± 18	357,969	53.3	63.3 ± 7.8*^f^*	0.0 (0/36)
2*^d^ (batch 2016a)^e^*	IHC	Feb-April 2016	2016	295 ± 22	348,341	57.3	50.1 ± 6.2	30.6 (11/36)
6 *(batch 6)^e^*	MRI	Jan-March 2018	2018	300 ± 28	368,715	50.4	26.9 ± 2.4	44.4 (16/36)
7 *(batch 7)^e^*	MRI	May-July 2018	2018	300 ± 27	369,535	51.2	31.7 ± 4.8	41.7 (15/36)

Table adapted from Liu et al. These batches of nPM have been reported from prior experiments (16).

PNC, particle number concentration; TOM, total organic matter; PM, particulate matter; IHC, immunohistochemistry; MRI, magnetic resonance imaging.

^a^Average PM values are the average DustTrack observations in the mid-way of each day of exposure. The variation (SD) through different days are less than 10% of the average values.

^b^SMPS records total PNC values for each nPM batch used in the beginning of each exposure.

^c^Total mass of Organic matter (OM) is reported by multiplying total organic carbon (TOC)% by a factor of 1.6 ± 0.2 recommended for urban aerosols to account for contributions of non-carbon atoms and the effect of PM oxygenation in the ambient through photochemistry (17).

^d^There were no exposure differences between Exposures 1 and 2.

^e^nPM exposure batch numbers represent those listed in Zhang et al. (18).

^f^Data variation is represented as standard deviation (SD).

**Table 2:** Mass fractions of organic carbon, trace elements, and metals during exposures.

Species	Exposure 1	Exposure 2	Exposure 6	Exposure 7
Total Organic Carbon (TOC)	395.6	312.9	168.1	198.0
S	37.68848	37.39218	26.24012	21.23598
Na	36.88259	36.25006	11.40668	45.62672
Ca	33.28122	30.25656	10.81711	43.26844
Mg	10.33886	8.29025	3.21959	12.87839
Al	8.84705	0.34266	9.84518	23.14781
Fe	8.64647	0.07821	3.47217	39.38073
K	6.67181	5.51256	6.70077	26.80310
Zn	2.98281	2.60414	0.59947	5.99470
P	0.98802	0.40049	0.44515	0.38551
Ba	0.74808	0.46081	0.00062	1.63285
Cu	0.58003	0.28747	0.26993	1.34965
Ti	0.34601	0.00653	0.17596	0.59326
Mn	0.33301	0.24310	0.05932	0.67426
B	0.30197	0.28301	0.13835	0.61619
Ni	0.25772	0.20169	0.04303	0.60265
Cr	0.16459	0.01917	0.03371	0.55343
Sb	0.12628	0.09871	0.15066	0.16709
Pb	0.11019	0.00492	0.00014	1.17308
Sn	0.06625	0.00847	0.00168	0.43030
Mo	0.05397	0.04402	0.00169	0.09037
Se	0.04485	0.03947	0.00504	0.00456
V	0.03795	0.02041	0.00359	0.14389
Li	0.01611	0.01131	0.01377	0.05508
Rb	0.0143	0.0056	0.01540	0.02658
As	0.01406	0.01044	0.00485	0.04032
Co	0.01186	0.00766	0.00154	0.06167
Ce	0.00678	0.00029	0.00347	0.02428
W	0.00499	0.00312	0.00041	0.00047
La	0.00394	0.00009	0.40821	0.00926
Cd	0.00369	0.00262	0.00065	0.01062
Nd	0.00257	0.00009	0.00060	0.00678
Y	0.00209	0.00016	0.01690	0.00674
Sc	0.001	0.00003	0.00055	0.00267
Nb	0.00082	0.00003	0.00077	0.00259
Cs	0.00078	0.00023	0.04177	0.00369
Ag	0.00076	0.00004	0.00022	0.00241
Pr	0.00067	0.00002	0.00664	0.00250
U	0.00059	0.00012	0.00066	0.00074
Th	0.00057	0.00002	0.27095	0.30347
Tl	0.00047	0.00041	0.00002	0.00002
Sm	0.00046	0.00002	0.00231	0.00139
Pd	0.00035	0.00013	0.00002	0.00116
Dy	0.00034	0.00002	0.00016	0.00018
Hf	0.00028	0.00003	0.00002	0.00002
Yb	0.00017	0.00002	0.00005	0.00006
Eu	0.00016	0.00003	0.00040	0.00058
Ho	0.00007	0.00000	0.00029	0.00033
Pt	0.00004	0.00003	0.00278	0.00318
Rh	0.00003	0.00002	0.02259	0.00021
Lu	0.00003	0.00000	0.00014	0.00016

Table adapted from Liu et al. These batches of nPM have been reported from prior experiments (16). Units are ng/μg of PM mass. The following elements are important for redox-active responses: transition metals Fe, Cu, Ti, Mn; Post transition: Al, Zn, Pb, Sn; Alkali and earth metals: Na, Ca, Mg, K; Non-metals: S, P, Se (19).

### Bilateral Carotid Artery Stenosis (BCAS) Surgery

BCAS procedures (11, 20), were performed 30 days prior to the conclusion of nPM/filtered air exposure (**Figure 1A**). Rectal temperature was maintained at 36.5-37°C. Mice were anesthetized by intraperitoneal ketamine/xylazine and placed in the prone position. Cerebral blood flow (CBF) values were monitored with a microtip fiber probe (Probe 418-1 master probe PF 5010 Laser Doppler Perfusion Monitoring Unit, Perimed AB, Sweden) fixed to the skull 1mm posterior and 5mm left of bregma. The mouse was then rotated to the supine position and bilateral common carotid arteries were exposed through a midline cervical incision. A 0.18 mm diameter microcoil (Sawane company, Japan) was applied to one carotid artery. CBF was recorded after the first coil application. A second microcoil was applied to the other carotid artery and CBF was recorded again. The incision was closed, and the mouse recovered in a temperature-controlled environment. For details of the surgeries see Liu et al. (16). Briefly, CBF drop right after insertion of microcoils was not different between filter+BCAS (35%) and nPM+BCAS (33%) groups. Total mortality during the study was 29.2% (42 out of 144 mice) and occurred only in the BCAS groups. No mice died in the filter and nPM groups. Mortality after BCAS surgery was not different between filter+BCAS (50%) and nPM+BCAS groups (52.5%, p=0.83).

### MRI

MRI scans were performed using a 7T PET-MR system (MR solutions Ltd, Guildford, UK) with a standard 20-mm internal diameter quadrature bird cage mouse head coil with a setup described in our earlier publications (21, 22). For *in vivo* scans the animals were anesthetized with 1.5-2.0% Isoflurane/air, cannulated for tail vein injections and monitored for respiration with an abdominal pressure-sensitive probe (80-100 BPM) and temperature with a rectal probe (36.5 ± 0.5°C) to ensure reproducible depth of anesthesia (Minerve mouse imaging cell, Esternay, France). The following sequences were used in this study: T2-w anatomical images (2D-fast spin echo (FSE), TR/TE 4,600/28 ms, 40 slices, slice thickness 500 μm, in-plane resolution 100x50 μm^2^), diffusion weighted echo-planar imaging (EPI, TR/TE 5,000/32, 14 slices, 1 b-value, 7 directions, slice thickness 300 μm, in-plane resolution 200×300 μm^2^), T1 mapping (1 slice fast low angle shot (FLASH) with variable flip angle, FA 5-45°, TR/TE = 20/4 ms, slice thickness 1 mm, in-plane resolution 60x120 μm^2^), T1-w dynamic contrast enhanced (DCE) MRI for BBB permeability (1 slice FLASH with identical geometry to T1 mapping, 180 images with temporal resolution of 5.1 s, FA 15°, TR/TE = 20/4ms) and T2*-w dynamic susceptibility contrast (DSC) MRI for CBF (1 slice FLASH, 80 images with temporal resolution of 1.4 s, FA 15°, TR/TE = 18/3ms, slice thickness 1 mm, in-plane resolution 120x230 μm^2^). Gadolinium-DTPA (Gd-DTPA) (Magnevist^®^, Bayer) diluted in saline was administered with a power injector during DCE (140μl, 1:4 dilution, 600μl/min) and DSC (140μl, 1:1 dilution, 1000μl/min) scans.

Analysis of DCE-MRI and DSC-MRI data was performed using Rocketship software (https://github.com/petmri/ROCKETSHIP) (23). Analysis of angiography images was done using ImageJ and otsu-thresholded maximum intensity projection (MIP) images and presented as relative blood flow referring to the signal intensity of vessels of interest in MIP images. Diffusion tensor imaging (DTI)-EPI parametric maps were generated using DSI Studio (March 6, 2018 build; dsi-studio.labsolver.org) as previously described (24). The diffusion weighted image data was pre-processed with motion, ghosting and eddy current corrections. After the co-registration of all individual images, data means were extracted for fractional anisotropy (FA) and apparent diffusion coefficient (ADC; x10^−3^ mm^2^/s) through DSI studio. Regions of interest (ROIs) for corpus callosum were manually assigned by an investigator blinded to groups. To examine distinct changes, regional ROIs were drawn on ADC maps. Due to time-constraints, six animals per group underwent MRI. One animal from filter+BCAS group was excluded from the study due to gray matter changes in T2-w images that indicated development of ischemic stroke. For DSC-MRI, one animal in the nPM+BCAS group was excluded due to motion artifact.

### Immunohistochemistry

Following the exposure period, mice were humanely euthanized with an intraperitoneal injection of ketamine and xylazine and transcardially perfused with PBS with 5 U/ml heparin followed by 4% paraformaldehyde in 0.01 mol/L PBS buffer. Brains were fixed in 4% paraformaldehyde for 24 hours at 4°C and stored in 70% ethanol. The tissue was paraffin embedded and 5 µm coronal sections were sliced from a part of the brain located from 1 mm anterior to 2 mm posterior to the bregma. Slides were deparaffined and hydrated using a series of alcohol dilutions (from 100% to 70% ethanol).

For myelin basic protein (MBP) and SMI-312 staining, antigen was retrieved with 10 mM sodium citrate and sections were blocked in 5% donkey serum in PBS with 0.3% Triton X-100 for one hour. Slides were incubated overnight at 4°C with primary antibody. Primary antibodies used were mouse anti-SMI-312 (BioLegend, 837904, 1:500) and rabbit anti-MBP (Santa Cruz Biotechnology, sc-13914-R, 1:200). Sections were then washed with PBS and incubated with secondary antibody for one hour. Secondary antibodies used were Alexa Fluor 568-donkey anti-mouse (Invitrogen; A-10037; 1:200) for SMI-312 and Alexa Fluor 568-donkey anti-rabbit (Invitrogen A; 10042; 1:200) for MBP. For IgG and Lectin double immunostaining, slides were washed in PBS and incubated with both Alexa Fluor 568-donkey anti-mouse IgG (Invitrogen; A-10037; 1:200) and Dylight 488-conjugated L. esculentum lectin (Vector Labs DL-1174; 1:200) for one hour. For all immunofluorescence, nuclei were stained with Hoechst 33342 (Life Technologies; 1:5000) for 10 minutes and sections were mounted with Agilent fluorescent mounting media. Slides were coverslipped and imaged at 400x using BZ-X810 fluorescent microscopy (Keyence, NJ). One slide per animal was assessed in each of the immunofluorescent stains (MBP, SMI312, and IgG and Lectin co-stain).

Sample sizes were n=12 per group for IgG and Lectin, n=12 for filter, nPM, and nPM+BCAS groups and n=11 for filter+BCAS group for SMI-312, and n=12 for filter, nPM, and nPM+BCAS groups and n=9 for filter+BCAS for MBP. All slides that underwent staining were analyzed and none were excluded.

Two blinded, independent observers analyzed all the sections, and their results were averaged. MBP and SMI-312 integrated fluorescence density was analyzed in both the right and left medial corpus callosum of one high-powered field (400x) and the two values were averaged. For quantification of extravascular IgG, IgG integrated density was analyzed outside of lectin-positive vascular profiles using Image J software (National Institutes of Health (NIH)) as we previously described (20). Briefly, IgG signal that colocalized with lectin-positive signal was subtracted from the total IgG immunofluorescence to obtain extravascular IgG integrated density. Extravascular IgG integrated fluorescence density was assessed in the right and left medial corpus callosum of one image (400x) and the results were subsequently averaged. All image analysis was done using Image J software (NIH) and followed the NIH Image J user guide.

### Statistical Analysis

Statistical analyses were carried out using GraphPad Prism (GraphPad Software LLC, version 9.0.0). The data is presented as mean ± standard error of the mean, mean ± standard deviation or violin plot with median and interquartile range as indicated in figure legends. Statistical testing was performed using 1-way or 2-way ANOVA followed by Bonferroni test. Datasets were screened for outliers using Grubb’s test and alpha = 0.05, but no outliers were detected. P-value below 0.05 was considered statistically significant.

## Results

### Blood Flow Is Reduced in the White Matter of BCAS Mice Exposed to nPM

To study the effects of combined cerebral hypoperfusion and nPM exposure, mice were exposed to nPM aerosol or filtered air for 6 weeks. Mice then underwent either bilateral carotid artery stenosis surgery or remained as controls (**Figure 1A**). After the surgeries, the nPM or filtered air exposure was continued for another 4 weeks (total exposure: ten weeks) before MRI and tissue collection (**Figure 1A**). The physiochemical properties of exposure aerosol are in **Figures 1B**–**G**. Measured average particulate matter mass concentration of the exposure aerosol was 310 µg/m^3^ (**Figure 1C**), total particulate number concentration 3.6 x 10^5^ particles/cm^3^ (**Figure 1D**), mode diameter 50 nm (**Figure 1E**), and total organic mass 43% total PM mass (**Figure 1F**). The elemental composition of the aerosol was further analyzed by inductively coupled mass spectrometry (ICP-MS), (**Figure 1G**). Overall, the exposure aerosol characteristics were typical for a sample collected from an urban area, where traffic-related emissions account for a major part of the air pollution profile, with organic matter (TOM) being the most predominant PM constituent and with an appreciable content of redox active metals and trace elements such as Fe, Mn, Ni, Cu and Zn (across nPM batches, **Table 2**) (25). The PM mass concentration, total particle number concentration, and the mode diameter were similar across nPM batches (**Table 1**).

**Figure 2:**
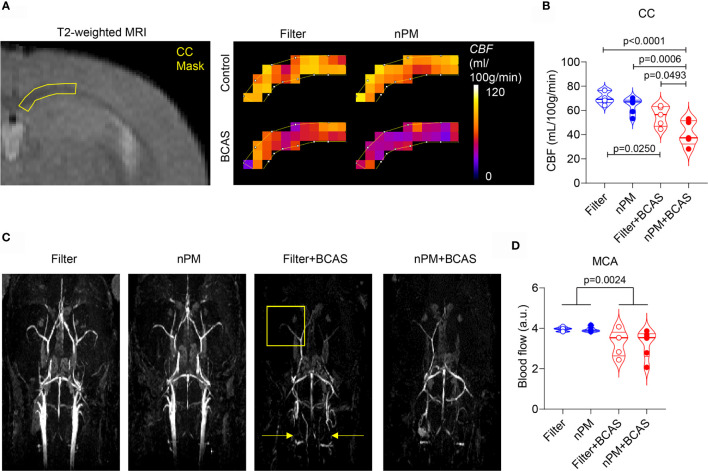
nPM exposure worsens white matter hypoperfusion after bilateral carotid artery stenosis. **(A, B)** Corpus callosum cerebral blood flow (CBF) was measured *in vivo* by using dynamic susceptibility weighted MRI and gadolinium-based contrast agent. Regional cerebral blood flow values (CBF in mL/100g/min) were mapped **(A)** and quantified **(B)** in the corpus callosum area. In panel **(B)** n=6 in filter and nPM groups and n=5 in filter+BCAS and nPM+BCAS groups. **(C)** Time-of-flight angiography was used to visualize arterial angioarchitecture in mice and is shown as maximum intensity projections. Yellow arrows indicate placement of microcoils around carotid arteries of hypoperfused mice and yellow square indicates the middle cerebral artery area, which was quantified in **(D)**. In panel **(D)** n=6 in filter, nPM and nPM+BCAS groups and n=5 in filter+BCAS group. P-value indicates BCAS effect in panel **(D)**. Data is presented as violin plot with median and quartiles. 1-way ANOVA **(B)** and 2-way ANOVA **(D)** and Bonferroni *Post Hoc* tests were used for statistical testing. CC, corpus callosum; MCA, middle cerebral artery.

After the completed exposure of 10 weeks, we first measured regional CBF values in the mice using DSC-MRI (**Figures 2A, B**). As expected, both filter+BCAS and nPM+BCAS groups had lower (by 21-42%) cerebral blood flow values compared to the filtered air group (**Figure 2B**). White matter hypoperfusion was further reduced by 26% in the nPM+BCAS group compared to filter+BCAS group. No effect was seen with nPM exposure alone in controls. The 3D time-of-flight (TOF) MR angiography images were analyzed for large vessel blood flow in the neck and brain (**Figures 2C, D**). Exposure to nPM had no effect on large caliber vessel blood flow either in control (filter *vs*. nPM) or BCAS operated (filter+BCAS *vs*. nPM+BCAS) mice, illustrated for the middle cerebral artery (MCA) in **Figure 2D**. However, BCAS effect reduced the blood flow signal in the MCA by 17-18% (p<0.0025). Taken together, these results indicate that nPM exposure worsens white matter microvascular blood flow in hypoperfused brain.

### nPM Exposure Increases White Matter BBB Leakage Caused by Hypoperfusion

**Figure 3:**
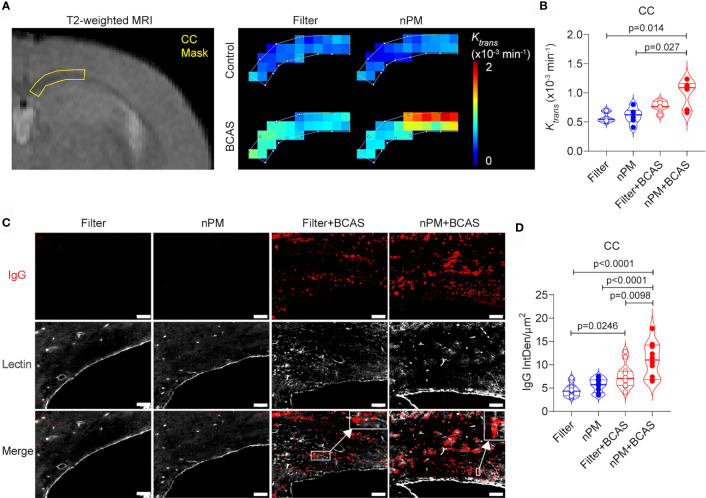
nPM exposure exacerbates white matter blood brain barrier leakage after bilateral carotid artery stenosis. **(A, B)** Corpus callosum blood brain barrier (BBB) permeability was measured *in vivo* by using dynamic contrast enhanced MRI. Distribution of gadolinium-based contrast agent was followed during the scan and regional BBB transfer constant (K_trans_, x10^-3^ min^-1^) was mapped **(A)** and quantified **(B)** in the corpus callosum area. In panel **(B)** n=6 in filter, nPM and nPM+BCAS groups and n=5 in filter+BCAS group. **(C)** BBB leakage was visualized postmortem by staining for extravascular IgG deposits. IgG positive deposits (red) outside lectin (white) positive blood vessels were quantified and presented as integrated density **(D)**. In panel **(D)** n=12 in all groups. Data is presented as violin plot with median and quartiles. 1-way ANOVA and Bonferroni *Post Hoc* tests were used for statistical testing. Scale bar = 50 µm in panel **(C)**. CC, corpus callosum; nPM, nanoparticulate matter; IgG, Immunoglobulin G; IntDen, integrated density.

We have previously shown that BCAS leads to BBB breakdown in mouse white matter (20), thus we used DCE-MRI to measure the BBB permeability in the corpus callosum of the mice *in vivo* (**Figures 3A, B**). Based on the quantification of *K_trans_* values, the combination of nPM exposure and BCAS caused a major BBB breakdown (69% permeability increase for a <1 kDa Gd-contrast agent) in the white matter of mice (**Figure 3B**). nPM exposure alone did not affect white matter BBB permeability values measured by DCE-MRI. Postmortem studies confirmed these results by immunohistochemistry for blood-derived IgG (~150 kDa, **Figures 3C, D**). IgG signal was negligible in brains of control mice with filtered air or nPM exposure (**Figure 3D**). Remarkably, quantification of IgG signal outside lectin-positive blood vessels revealed heavy deposition of IgG in the white matter of nPM exposed BCAS mice (140% increase compared to filtered air) confirming the DCE-MRI results. In addition, the IgG deposition was 44% higher in the nPM+BCAS group compared to the filter+BCAS group. These results demonstrate that nPM exposure aggravates BBB breakdown induced by hypoperfusion in the white matter of mice.

### MRI Diffusion Metrics Are Abnormal in the White Matter After Combined BCAS and nPM Exposure

**Figure 4:**
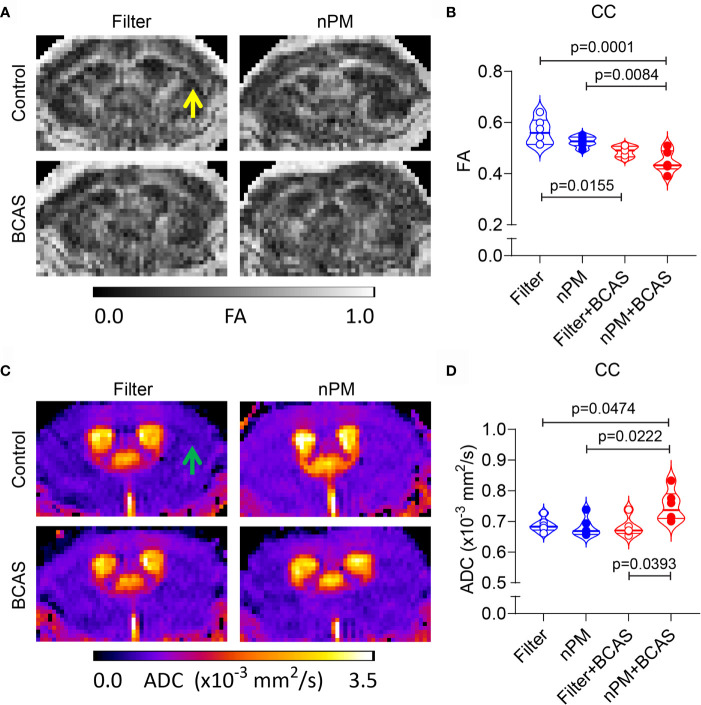
Altered diffusion metrics in white matter of nPM exposed BCAS mice. **(A, B)** Diffusion weighted MRI was used to measure integrity of white matter in mice and FA maps were generated **(A)** and quantified within the corpus callosum area **(B)**. **(C, D)** ADC maps were generated using diffusion weighted MRI **(C)** and values (x10^-3^ mm^2^/s) were quantified in the corpus callosum area **(D)**. Notice lack of white and gray matter contrast especially in nPM+BCAS group in the corpus callosum area [arrows in **(A, C)**]. In panels **(B, D)** n = 6 in filter, nPM and nPM+BCAS groups and n = 5 in filter+BCAS group. Data is presented as violin plot with median and quartiles. 1-way ANOVA and Bonferroni *Post Hoc* tests were used for statistical testing. CC, corpus callosum; FA, fractional anisotropy; ADC, apparent diffusion coefficient.

Chronic cerebral hypoperfusion induced by BCAS causes white matter damage in mice (11). We sought to examine whether diffusion-weighted MRI would reveal changes in the white matter diffusion metrics, which we showed in other models of vascular white matter injury (21). First, we measured the fractional anisotropy (FA) values in the corpus callosum (**Figures 4A, B**). We noted that both BCAS surgery alone and BCAS surgery combined with nPM exposure significantly reduced FA values in the corpus callosum (by 14 and 21%) compared to the filtered air group (**Figure 4B**). nPM exposure without BCAS did not affect FA values. Moreover, apparent diffusion coefficient values (ADC) were higher (by 10%) in the nPM+BCAS group compared to all other groups (p< 0.05) (**Figure 4C, D**). We did not detect changes in white matter ADC values in mice that underwent nPM exposure or BCAS surgery alone. Altogether, BCAS led to abnormal diffusion metrics, suggestive of white matter structural damage, with abnormalities further worsened by nPM exposure.

### Demyelination After BCAS Is Aggravated by nPM Exposure

**Figure 5:**
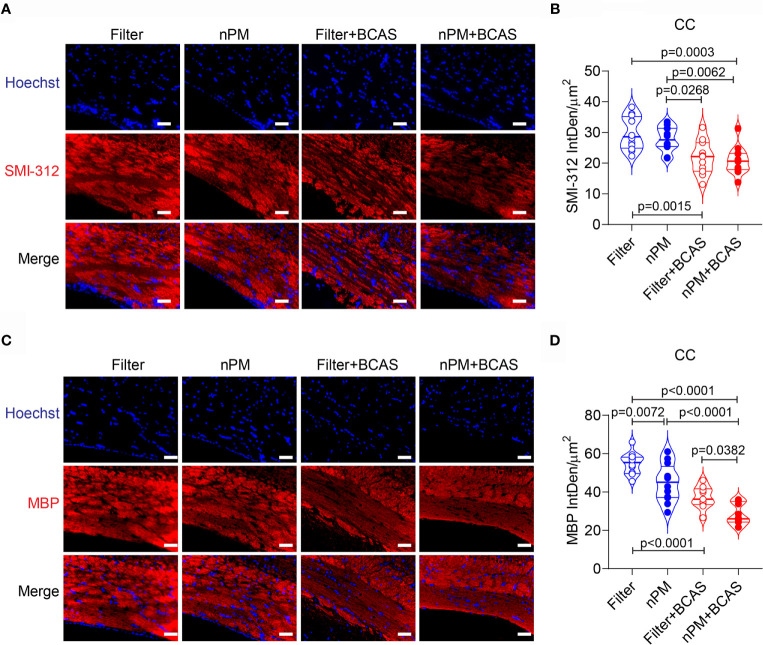
Increased demyelination in white matter of nPM exposed mice. **(A)** White matter axons were visualized by staining with SMI312 antibody (red) and quantified in the corpus callosum area **(B)**. In panel **(B)** n=12 in filter, nPM and nPM+BCAS groups and n=11 in filter+BCAS group. **(C)** White matter myelin was visualized by staining with myelin basic protein (red) antibody and quantified in the corpus callosum area **(D)**. In panel **(D)** n=12 in filter, nPM and nPM+BCAS groups and n=9 in filter+BCAS group. Data is presented as violin plot with median and quartiles. 1-way ANOVA and Bonferroni *Post Hoc* tests were used for statistical testing. Scale bars = 50 µm in panels **(A, C)**. CC, corpus callosum; IntDen, integrated density; MBP, myelin basic protein.

To study whether altered diffusion metrics in the white matter were linked to axonal damage or demyelination, we stained brain sections collected from all groups for SMI-312 (axonal marker) and myelin basic protein (MBP) (**Figures 5A**–**D**). As expected (26), axonal density in the corpus callosum was reduced in both BCAS groups (by 27 and 30%) compared to filtered air group, (**Figures 5A, B**). However, no nPM effect was seen either in control (filter *vs*. nPM) or BCAS groups (filter+BCAS *vs*. nPM+BCAS; **Figure 5B**). Finally, myelin density in the corpus callosum was decreased in both nPM (18%) and nPM+BCAS (48%) groups compared to the filtered air group (**Figure 5D**). Demyelination was also observed in the filter+BCAS group (32% compared to filtered air group), but nPM exposure decreased the myelin density by an additional 24% when combined with BCAS (nPM+BCAS *vs*. filter+BCAS; **Figure 5D**). These results suggest that BCAS reduces both axonal density and myelination in the white matter in mice, whereas nPM exposure primarily leads to demyelination.

## Discussion

We have demonstrated that air pollution nPM exposure worsens white matter blood flow and increases BBB permeability in mice with chronic cerebral hypoperfusion using translational multiparametric MRI and postmortem histological confirmation. While nPM exposure alone did not alter regional cerebral blood flow or BBB integrity, the combined nPM+BCAS cohort demonstrated decreased CBF values and increased BBB permeability (extravascular IgG deposits) when compared to the filter+BCAS mice. Moreover, disturbances in diffusion metrics in the corpus callosum region were detected in the combined nPM+BCAS exposure group using diffusion-weighted MRI. Immunohistochemical staining of corpus callosum axons and myelin clearly demonstrated that chronic cerebral hypoperfusion leads to axonal degeneration and demyelination and that the demyelination was further exacerbated by nPM exposure. Notably, nPM exposure led to demyelination in control mice even in the absence of hypoperfusion.

Murine BCAS results in subtle, chronic hypoperfusion that leads to structural and functional white matter damage and neuroinflammation over a 30-day time course (11, 27). The temporal changes in CBF values in the mouse BCAS model have been previously documented using doppler (11) and arterial spin labeling MRI (28, 29). Here, we applied DSC-MRI techniques that utilize tracking of a bolus of contrast agent through the brain vasculature to map and measure regional blood flow values. We have previously shown the utility of this method for detecting hypoperfusion in mouse white matter (21). The BCAS effect on white matter blood flow values was prominent in both filtered air and nPM exposed groups. Interestingly, the hypoperfusion was worse in the white matter of the combined nPM+BCAS exposure cohort when compared to BCAS alone (filter+BCAS). When TOF MR angiography was used to visualize the large vessel blood flow in the brain, we did not see a difference between the two BCAS groups. Considering that the white matter vasculature consists principally of capillaries (30), these results suggest that the nPM exposure amplified the microcirculatory damage that was caused by CCH.

Our previous murine studies have shown that nPM exposure elicits neuroinflammation in the brain white matter. Mice exhibit reactive microglia and increased deposition of complement C5 protein in the corpus callosum following 150 hours of nPM exposure (31). Stroke studies demonstrate markers of inflammation (complement C5, C5a receptor) and oxidative stress (gp91^Phox^, 8 hydroxyguanasine) in the region of the ischemic penumbra after 45 cumulative hours of nPM exposure (32). Further, studies have demonstrated that ultrafine particulate matter induces TNFα production and activates microglia, which inhibit neurite outgrowth and decrease Myelin Basic Protein in the hippocampal CA1 region (10). Increased BBB permeability may amplify microvascular failure resulting from neuroinflammation and modulate the nPM-induced corpus callosum white matter injury.

BBB breakdown is a common finding in aging and demented human brains (33, 34) and predicts cognitive decline in Alzheimer's disease (AD) (34, 35). We have previously shown that BBB breakdown occurs in the murine BCAS model (20) and contributes to the white matter damage in a mouse model of pericyte deficiency (14). Based on our DCE-MRI results in the current study, we found that the nPM exposure led to a severe BBB breakdown in the BCAS cohort that was not evident in the nPM (control) group. In line with the findings of DCE-MRI that utilizes a small molecular weight gadolinium-based contrast agent, the results were confirmed with IgG staining in the corpus callosum white matter. IgG is typically restricted to the vascular compartment and cannot be detected in the white matter parenchyma as we show in the control animals with filtered air or nPM exposure alone. However, the BBB opening induced by cerebral hypoperfusion allowed IgG to extravasate particularly when the hypoperfusion was combined with nPM exposure. Importantly, IgG extravasation into the white matter signifies that other blood-derived molecules such as fibrinogen, albumin, and thrombin can also enter the white matter and elicit toxic effects (36). Compromised BBB integrity in the nPM+BCAS group supports the findings of decreased CBF measurements, highlighting the susceptibility of the capillary network to this combined exposure.

Diffusion MRI is widely used in human white matter disease studies (37). We show here that the combined nPM+BCAS exposure leads to abnormalities in white matter diffusion metrics that are less prominent in the filter+BCAS group. Our findings of reduced white matter FA and increased ADC values are in line with our previous studies (21) and clinical reports (37) on white matter disease of vascular origin. The findings also match previous reports in the mouse BCAS model itself, showing reduced corpus callosum FA values without changes in the mean diffusivity (38). The validity of these findings was confirmed by post-mortem analysis of axonal and myelin density in the corpus callosum area. BCAS itself led to both axonal degeneration and demyelination, with more severe demyelination in the combined nPM+BCAS group. In this group, the demyelination could be linked to reduced white matter blood flow and deposition of blood-derived molecules (21). However, the fact that demyelination was independent of vascular dysfunction in the nPM (control) group suggests an autonomous toxic effect of nPM on oligodendrocytes.

The results of this study should be interpreted within the framework of the reported decreases in potency of recent Los Angeles nPM samples (18). Histochemistry studies were performed on mice exposed to nPM batches collected in 2015 and 2016, while MRI studies were performed on mice exposed to nPM batches collected in 2018 (batch 6,7). According to Zhang et. al, the 2018 nPM batches demonstrated decreased potency compared to the prior samples (18). Independent nPM effects were not strong in the current experiments regardless of nPM batch exposure. Only MBP densities differed between the filtered air and nPM cohorts (no changes in axonal integrity, diffusion metrics, BBB leakage, or white matter hypoperfusion). In contrast, the joint effects of the nPM and BCAS exposures are clear on multiple MRI and histochemical analyses. Exposure to nPM, even with potentially decreased potency, worsens cerebrovascular dysfunction and white matter injury in the setting of CCH. This indicates an interaction between these exposures that can yield supra-additive effects; a relationship has been demonstrated in Liu et al. (16).

Strengths of our study include utilization of air pollution nPM collected from a densely populated metropolitan area impacted by traffic emissions that are typical of major urban areas in the US (25) and MRI techniques (12), both increasing the translational potential of our findings. The set-up of this study’s nPM exposure system minimized within-exposure variability compared to other systems, such as particle concentrators. The particle composition of the exposure aerosol retained the chemical properties of traffic-related air pollution. Differences between nPM batches are representative of the natural variability of Los Angeles aerosol, as nPM was collected across different seasons and years. A potential limitation of our exposure system includes that re-suspended particles may not maintain the original particles’ surface chemistry or shape. Our study was limited to young male mice only and sex effect is known to exist in the mouse BCAS model (39). As filter mice served as controls for the IgG stain, unspecific IgG binding may be present in all groups. However, significant increases of extravascular IgG were demonstrated in the BCAS groups compared to filtered air. Lectin selectivity for vasculature may be impacted during disease. To minimize the effect of unspecific lectin binding on the quantification of extravascular IgG, IgG was quantified outside of lectin-positive vascular profiles using Image J software. The main purpose of this study was to characterize the outcome resulting from combined cerebral hypoperfusion and exposure to nPM using vascular and structural imaging. Further studies are needed to reveal the toxic components of nPM used in this study, and their effects on different cell types of neurovascular unit.

## Conclusion

In conclusion, we have shown that nPM exposure, when combined with CCH worsens cerebrovascular dysfunction and increases white matter demyelination. The results highlight the potential susceptibility of brain white matter to effects of nPM in the presence of underlying vascular disease.

## Data Availability Statement

The raw data supporting the conclusions of this article will be made available by the authors, without undue reservation.

## Ethics Statement

The animal study was reviewed and approved by Institutional Animal Care and Use Committee (IACUC) at the University of Southern California.

## Author Contributions

MH, QL, KS, MC, and AM, contributed to methodology. MH, QL, KS, MC, and KL-F contributed to data curation. MH, QL, KS, MC, KL-F, AP, AM and HB were involved in the investigation process. MH, QL, and MC, completed the formal analysis. MH, CS, TM, CF, and WM contributed to the conceptualization of this project. CS, TM, CF, BZ, and WM contributed to funding acquisition. CS, TM, CF, BZ, and WM supervised the project. MH, KS, MC, KL-F drafted the original manuscript. MH, CS, TM, CF, BZ, and WM drafted the final manuscript with critical review and revision. All authors contributed to the article and approved the submitted version.

## Funding

This study was supported by National Institutes of Health (NIH) grants 5R01ES024936-05 to WJM, 5R01NS100459-03 to BZ and 5P01AG055367 to WJM, CEF, CS, BZ. The funders had no role in study design, data collection and analysis, manuscript preparation, or decision to publish.

## Conflict of Interest

WM is a consultant for Rebound Therapeutics, Viseon, Imperative Care, Integra, Q’Apel, Stryker, Stream Biomedical, Spartan Micro. WM is an investor for Cerebrotech, Endostream, Viseon, Rebound, Spartan Micro, Truvic, Imperative Care, Q’Apel.

The remaining authors declare that the research was conducted in the absence of any commercial or financial relationships that could be construed as a potential conflict of interest.

## Publisher’s Note

All claims expressed in this article are solely those of the authors and do not necessarily represent those of their affiliated organizations, or those of the publisher, the editors and the reviewers. Any product that may be evaluated in this article, or claim that may be made by its manufacturer, is not guaranteed or endorsed by the publisher.
